# What does early initiation and duration of breastfeeding have to do with childhood mortality? Analysis of pooled population-based data in 35 sub-Saharan African countries

**DOI:** 10.1186/s13006-021-00440-x

**Published:** 2021-12-07

**Authors:** Michael Ekholuenetale, Amadou Barrow

**Affiliations:** 1 Department of Epidemiology and Medical Statistics, Faculty of Public Health, College of Medicine, University of Ibadan, Ibadan, Nigeria; 2 Department of Public & Environmental Health, School of Medicine & Allied Health Sciences, University of The Gambia, Kanifing, The Gambia

**Keywords:** Childhood mortality, Optimal breastfeeding, Infant, Early breastfeeding initiation, DHS

## Abstract

**Background:**

Breastfeeding practices and their impact on infant health and survival are unquestionably of global interest. The aim of this study was to examine the link between breastfeeding initiation within one hour of birth, breastfeeding duration and childhood mortality in sub-Saharan Africa.

**Methods:**

This study used data from the Demographic and Health Survey, which was conducted in 35 Sub-Saharan African countries between 2008 and 2017. Early initiation and duration of breastfeeding, food consumption indices, and infant mortality were all important variables. Analysis used percentage, median/interquartile range, and regression models (logistic, linear, Cox).

**Results:**

Early initiation of breastfeeding within one hour after birth was lowest in Chad (23.0%) and highest in Burundi (85.0%). The pooled median duration of breastfeeding was 12 months. Female children had 3% significant lower odds of consuming tinned, powdered or fresh milk, compared with male children (OR 0.97; 95% CI 0.94, 0.99). Conversely, female children were more likely to be put to breast within one hour after birth, compared with male children (OR 1.03; 95% CI 1.01, 1.05). Results from the pooled sample showed approximately 20% (HR 0.80; 95% CI 0.67, 0.96) and 21% (HR 0.79; 95% CI 0.77, 0.80) reduction in infant mortality for children breastfed within one hour after birth and for every unit increase in the months of breastfeeding respectively. In addition, countries with the leading infant mortality rate include; Sierra Leone (92 deaths per 1000 live births), Chad (72 deaths per 1000 live births), Nigeria (69 deaths per 1000 live births), Cote d’ Ivoire (68 deaths per 1000 live births), Guinea (67 deaths per 1000 live births), Burkina-Faso (65 deaths per 1000 live births) and Mozambique (64 deaths per 1000 live births) respectively.

**Conclusions:**

The findings from this study underscores the need for early breastfeeding initiation and prolong breastfeeding to be considered in programmes on improving childhood survival. Efforts should be made to improve optimal breastfeeding practices as only about half of children in the pooled sample had best practices of breastfeeding.

## Background

To enable countries in Sub-Saharan Africa (SSA) to reach the third Sustainable Development Goal (SDG-3) of ending preventable deaths of newborns and children under the age of five, optimal feeding habits for newborns, infants, and children are a must [1]. Infant and child feeding practices have a considerable impact on the state of children’s nutritional and health condition, and are major contributors to child survival. As a result, encouraging newborn and child feeding behaviors is critical for enhancing children’s nutrition, health, and development [2]. World Health Organization (WHO) has recently released a set of indicators to assess child feeding patterns and track the effectiveness of breastfeeding advancement efforts. Since then, the newborn and child feeding structure, as well as knowledge of what constitutes good breastfeeding and supplementary feeding practices, have received a lot of attention [2]. Until now, the majority of the indicators employed in population surveillance systems to estimate newborn and child feeding behaviors have focused on breastfeeding [2, 3]. The indications show whether or not complementary foods are ingested, but not the quality or quantity of those foods.

Approximately half of all infants are breastfed during the first hour of their lives [4]. Breastfeeding is most common at 12 months in SSA, South Asia, and areas of Latin America [4]. Breastfeeding should begin during the first hour of life, be supplemented with nutritionally adequate foods, and be continued until at least two years of age, according to the WHO standard for infant and child feeding [2, 5]. Evidence-based studies have shown that early initiation of breastfeeding is linked to significant gains and improved childhood survival, bolstering the recommendation to begin breastfeeding as soon as possible after childbirth [6, 7]. Despite all of the benefits that have been revealed [8], in numerous resource-constrained contexts, rates of early initiation of breastfeeding still need to be improved in order to reap the benefits of proper feeding habits during early infancy. It is critical to identify characteristics linked to childhood feeding patterns in order to build effective nutrition programs and target the core demographic for optimal feeding practices.

Understanding breastfeeding patterns is critical for prioritizing knowledge gaps in the field of childhood survival [9]. Breastfeeding promotion is an important component of child survival treatments, and attempts have been made around the world to enhance newborn and child feeding patterns, such as the International Code of Marketing of Breast Milk Substitutes [10], Baby Friendly Hospital Initiative (BFHI) [11], and Global Strategy for Infant and Young Child Feeding [12]. Infants who receive adequate supplemental feeding grow faster and have fewer health problems than those who do not obtain adequate nutrition [13]. The demands of SSA’s newborns in terms of health and nutrition are critical, and optimal child growth and development are critical for driving the economic development process. Breastfeeding and optimal feeding assist newborns in a variety of ways, including delivering nutrients that help with growth and immunity [14]. There have been significant advances in child survival over the last three decades, while there are inequalities in these gains within and across countries [15]. Between 1990 and 2013, the Millennium Development Goals (MDGs) rallied global efforts to promote child survival, resulting in a half-decade reduction in child mortality [16]. More specifically, initiatives to improve childhood survival in the SSA region resulted in a fall in mortality rates from 180 per 1000 live births in 1990 to 83 per 1000 live births in 2015 [17]. Nonetheless, the MDGs may have failed to monitor key factors associated with childhood mortality, and resources may have been ineffectively deployed toward the complex factors associated with childhood mortality, resulting in SSA continuing to account for a large share of poor childhood survival around the world.

Data on childhood survival demonstrates that there is still more work to be done in SSA nations to improve children’s health. Breastfeeding and nutrition for infants, as well as vaccination against some of the worst childhood diseases such as pneumonia, polio, and measles, can all help to enhance childhood survival [18–20]. Children can also be protected from morbidity and mortality with proper sanitation and hygiene. High parental educational attainment, adequate social economic position, and closeness to health services, are socioeconomic characteristics that can promote childhood survival [21, 22]. Furthermore, growing research demonstrates that community-based approaches to improve child health in African countries are effective. Across SSA, community health workers are being deployed to provide timely healthcare, particularly to children in rural and isolated locations [23, 24]. According to a recent modeling study, child health treatments done in collaboration with communities have the potential to avoid 4.9 million deaths [25].

Evidence based on local data, is critical in advocating for targeted interventions to improve optimum newborn and child feeding practices by providing timely, culturally relevant, and context-specific information. Male-female disparities in childhood mortality estimates from numerous locations in SSA are persistently obvious, according to existing evidence [26]. In light of the above, we have endeavored to examine the association between breastfeeding practices and childhood mortality.

## Methods

### Data source

We used multi-country nationally representative DHS data from 35 SSA nations in this research. Data on all children born in the five years leading up to the surveys was pooled to create a sample of 384,747 births in selected SSA countries between 2008 and 2017. The data is in the public domain and was accessed at DHS website. DHS were based on a stratified multi-stage cluster sampling technique. Structured interviewer-administered questionnaires were used to obtain data on critical reproductive health issues. The maternity history, in which women were asked about their birth histories, was an important component of the data gathering. Data from the birth histories was recoded into individual records for each child listed by the mothers, with data on the research variables retrieved for analysis. Thirty-five countries in SSA region were included based on availability of recent data collected. These are: Angola, Benin, Burkina-Faso, Burundi, Cameroon, Chad, Comoros, Congo, Cote d’Ivoire, DR Congo, Ethiopia, Gambia, Gabon, Ghana, Guinea, Kenya, Lesotho, Liberia, Madagascar, Malawi, Mali, Mozambique, Namibia, Niger, Nigeria, Rwanda, Sao Tome & Principe, Senegal, Sierra Leone, South Africa, Tanzania, Togo, Uganda, Zambia and Zimbabwe.

### Measurement and description of variables

#### Months of breastfeeding

Months of breastfeeding was obtained based on mothers’ self-reported data. This was a continuous variable and women who were categorized; ever breastfed, not currently breastfeeding, never breastfed, inconsistent and don’t know were dropped from the variable.

#### Time of breastfeeding initiation

Time after the birth at which the respondent first breastfed the child. The dichotomous variable was based on whether the child was breastfed immediately after birth (within one hour) vs. whether breastfeeding was initiated after one hour.

#### Items of food consumption

Gave child plain water: yes/no; Gave child juice: yes/no; Gave child tinned, powdered or fresh milk: yes/no; Gave child infant formula: yes/no; Gave child fortified baby food (cerelac etc.): yes/no; Gave child other liquid: yes/no. These questions were answered for all infants.

#### Child characteristics

Childhood mortality: the death of a child aged one year. The data used for estimating infant mortality were based on all births in the five years preceding data collection [27]; Sex of child: male/female; Survival status: dead/alive.

#### Maternal and household factors

Mother’s age (years): 15–19, 20–24, 25–29, 30–34, 35–39, 40–44, 45–49; Mother’s educational level: No education, primary, secondary, higher; Place of residence: urban vs rural; Household wealth quintile: The wealth indicator weights were assigned using principal components analysis (PCA) for the production of the wealth index. The wealth indicator variables such as floor type, wall, roof, water supply, sanitation facilities, radio, electricity, television, refrigerator, cooking fuel, furniture, and number of persons per room were scored and standardized using this approach. After that, the z-scores and factor coefficient scores (factor loadings) were determined. Finally, the indicator values were multiplied by the loadings and totalled to obtain the wealth index value for each family. The standardized z-score was used to disentangle the overall assigned scores to poorest, poorer, middle, richer and richest [28].

### Ethical consideration

This study relied on DHS data that was made publicly available. Prior to the survey, participants gave their informed consent. The DHS Program adheres to industry norms for preserving the privacy of responders. ICF International verifies that the survey conforms with the regulations of the US Department of Health and Human Services regarding the protection of human subjects’ rights. The researchers received approval from DHS to use the de-identified secondary dataset for this study.

### Statistical analysis

Stata 14.0 was used to analyze the data (StataCorp, College Station, TX). Clustering (enumeration areas) and sampling weights were adjusted using the Survey (‘svy’) command. The prevalence of breastfeeding within one hour after birth was calculated using percentages. The summary statistics for months of breastfeeding was calculated using median and interquartile range (IQR). Logistic and linear regression were used to model patterns of breastfeeding and indicators of complementary food consumed. Cox regression was used to model childhood mortality. The probability of childhood death was modelled using the following equation;

$$ In\frac{h(t)}{h_0(t)}={b}_1{x}_1+{b}_2{x}_2+\dots +{b}_k{x}_k $$

Where *x*_*1*_ to *x*_*k*_ represent the explanatory variables and h_0_(t) is the baseline hazard at time t, representing the hazard for a child with zero values for all the explanatory variables. Thus, the dependent variable in the model is the log HR. The coefficients *b*_*i*_ to *b*_*k*_, which represent the effect of each explanatory variable, are estimated in the process of modelling. All statistical tests were conducted at 0.05 level of significance.

## Results

Table 1 shows disparities in the patterns of early initiation and duration of breastfeeding across 35 SSA countries. The median months of breastfeeding was lowest in Gabon (7.0), Congo and Namibia (8.0) respectively; and highest in Kenya and Sao Tome & Principe (15.0), Madagascar (16.0) and Zambia (18.0) respectively. In addition, early initiation of breastfeeding i.e., within one hour after birth was lowest in Chad (23.0%), Guinea (16.6%) and Congo (23.8%); and highest in Ethiopia (73.3%), Rwanda (80.5%) and Burundi (85.0%). Furthermore, total infant mortality rates were highest in Sierra Leone (92 deaths per 1000 live births), Chad (72 deaths per 1000 live births), Nigeria (69 deaths per 1000 live births), Cote d’ Ivoire (68 deaths per 1000 live births), Guinea (67 deaths per 1000 live births), Burkina-Faso (65 deaths per 1000 live births) and Mozambique (64 deaths per 1000 live births) respectively. See Table 1 for details.

**Table 1:** Distribution of breastfeeding practices and infant mortality rate in SSA countries; DHS 2008–2017

Country	Study year	Sample size (***n***)	Median months of breastfeeding (IQR)	Child put to breast within 1 h after delivery (%)	Infant mortality/1000 live births (95% CI)
Angola	2015/16	14,322	9.0 (11)	48.3	44 (39, 50)
Benin	2012	13,407	11.0 (12)	50.4	42 (38, 46)
Burkina-Faso	2010	15,044	12.0 (13)	42.1	65 (60, 70)
Burundi	2016/17	13,192	13.0 (13)	85.0	47 (42, 52)
Cameroon	2011	11,732	14.0 (9)	39.8	62 (56, 68)
Chad	2014/15	18,623	10.0 (12)	23.0	72 (66, 78)
Comoros	2012	3149	9.0 (11)	33.7	36 (27, 45)
Congo	2011/12	9329	8.0 (9)	23.8	39 (33, 46)
Cote d’Ivoire	2011/12	7776	10.0 (10)	30.8	68 (59, 77)
Democratic Republic of Congo	2013/14	18,716	11.0 (12)	51.9	58 (53, 63)
Ethiopia	2016	10,641	12.0 (14)	73.3	48 (41, 55)
Gambia	2013	8088	10.0 (10)	51.5	34 (28, 40)
Gabon	2012	6067	7.0 (8)	32.3	43 (35, 50)
Ghana	2014	5884	11.0 (12)	55.6	41 (34, 48)
Guinea	2012	7039	12.0 (12)	16.6	67 (58, 75)
Kenya	2014	20,964	15.0 (14)	62.2	39 (35, 43)
Lesotho	2014	3138	10.0 (11)	65.3	59 (49, 70)
Liberia	2013	7606	10.0 (11)	61.2	54 (46, 61)
Madagascar	2008/09	12,448	16.0 (13)	70.7	48 (43, 53)
Malawi	2015/16	17,286	12.0 (12)	76.3	42 (38, 46)
Mali	2012/13	10,326	11.0 (13)	57.8	56 (49, 63)
Mozambique	2011	11,102	10.0 (11)	76.7	64 (58, 71)
Namibia	2013	5046	8.0 (10)	71.2	39 (32, 46)
Niger	2012	12,558	10.0 (10)	52.9	51 (45, 56)
Nigeria	2013	31,482	9.0 (10)	33.2	69 (64, 73)
Rwanda	2014/15	7856	14.0 (16)	80.5	32 (28, 37)
Sao Tome & Principe	2008/09	1931	15.0 (9)	42.9	38 (28, 48)
Senegal	2017	12,185	10.0 (11)	33.6	42 (37, 46)
Sierra Leone	2013	11,938	10.0 (11)	53.8	92 (85, 100)
South Africa	2016	3548	9.0 (13)	67.3	35 (26, 44)
Tanzania	2015/16	10,233	10.0 (11)	51.2	43 (38, 48)
Togo	2013/14	6979	12.0 (12)	60.6	49 (42, 55)
Uganda	2016	15,522	10.0 (11)	66.1	43 (39, 47)
Zambia	2013/14	13,457	18.0 (9)	65.8	45 (40, 49)
Zimbabwe	2015	6132	9.0 (10)	57.6	50 (44, 56)

Figures in brackets in column 4 indicate Interquartile range (IQR)

The pooled median (IQR) duration of breastfeeding was 12.0 months. In addition, the pooled breastfeeding within one hour after birth was 55.1%. Table 2 shows the indices of childhood food consumption and the patterns of breastfeeding at the aggregate level and by gender in SSA. The feeding of plain water was high in SSA (81.4%) with no differences between male and female child. In addition, feeding of juice, infant formula, fortified baby food (cerelac, etc.), tinned, powdered or fresh milk and other liquid were low in SSA. See Table 2 for details.

**Table 2:** Feeding of complementary food items consumed and breastfeeding practices in SSA; DHS 2008–2017

Variable	Pooled sample			Male			Female		
	***n***	%	Median (IQR)	***n***	%	Median (IQR)	***n***	%	Median (IQR)
**Index of Child’s Food Consumption**									
Gave child plain water	239,373	81.4		120,906	81.5		118,467	18.7	
Gave child juice	227,968	9.1		115,059	9.1		112,909	9.1	
Gave child tinned, powdered or fresh milk	239,313	12.5		120,859	12.6		118,454	12.4	
Gave child infant formula	239,050	4.6		120,727	4.6		118,323	4.6	
Gave child fortified baby food (cerelac etc.)	220,760	7.3		111,586	7.3		109,174	7.2	
Gave child other liquid	238,988	19.6		120,704	19.6		118,284	19.7	
**Duration of child put to breast after delivery**									
Within 1 h	135,222	55.1		68,002	54.8		67,220	55.4	
After 1 h	110,193	44.9		56,052	45.2		54,141	44.6	
**Type of breastfeeding for child**									
**Months of breastfeeding**	160,714		12.0 (12)	80,963		12.0 (12)	79,751		12.0 (12)

Table 3 examined whether female children were given the same food items as male children in the preceding 24 h to the survey and the differentials in the patterns of breastfeeding between female vs. male children. After adjusting for age, results showed that female children had a 3% significant reduction to consume tinned, powdered or fresh milk, compared with male children (OR 0.97; 95% CI 0.94, 0.99). Conversely, female children were 1.03 times as likely to be put to breast within one hour after birth, compared with male children (OR 1.03; 95% CI 1.01, 1.05).

**Table 3:** Differences in breastfeeding practices and food items consumption between females and males; DHS 2008–2017

Variable	Exp (Coef.)	Odds ratio	95% CI	***P***-value
Gave child plain water		0.98	0.95, 1.00	0.069
Gave child juice		1.02	0.98, 1.06	0.445
Gave child tinned, powdered or fresh milk		0.97	0.94, 0.99***	0.050
Gave child infant formula		0.99	0.94, 1.04	0.788
Gave child fortified baby food (cerelac etc.)		0.96	0.93, 1.01	0.088
Gave child other liquid		1.00	0.98, 1.03	0.735
Child put to breast within 1 h after delivery		1.03	1.01, 1.05***	0.006
Months of breastfeeding	1.02		0.95, 1.10	0.606

***significant at *p* < 0.05; Adjusted for age

Table 4 showed the effect of breastfeeding patterns on childhood survival. Results from the pooled sample showed approximately 20% (HR 0.80; 95% CI 0.67, 0.96) and 21% (HR 0.79; 95% CI 0.77, 0.80) reduction in the risk of infant mortality for children breastfed within one hour after birth and for every unit increase in the months of breastfeeding respectively. Similarly, male children had 24% (HR 0.76; 95% CI 0.59, 0.97) and 22% (HR 0.78; 95% CI 0.76, 0.80) reduction in the risk of infant mortality for children breastfed within one hour after birth and for every unit increase in the months of breastfeeding, respectively. In addition, female children had 20% (HR 0.80; 95% CI 0.77, 0.82) reduction in the risk of infant mortality for every unit increase in the months of breastfeeding, after adjusting for place of residence, maternal age and educational attainment and household wealth quintile.

**Table 4:** Effect of early initiation and duration of breastfeeding in infant mortality

Variable	Pooled sample				Male				Female			
	Unadjusted HR (95%CI)	***p***	Adjusted HR (95%CI)	***p***	Unadjusted HR (95%CI)	***p***	Adjusted HR (95%CI)	***p***	Unadjusted HR (95%CI)	***p***	Adjusted HR (95%CI)	***p***
Child put to breast within 1 h after delivery	0.84 (0.79, 0.88)	< 0.001	0.80 (0.67, 0.96)	0.016	0.83 (0.77, 0.89)	< 0.001	0.76 (0.59, 0.97)	0.028	0.84 (0.78, 0.91)	< 0.001	0.85 (0.65, 1.12)	0.250
Duration of breastfeeding	0.81 (0.80, 0.82)	< 0.001	0.79 (0.77, 0.80)	< 0.001	0.80 (0.79, 0.81)	< 0.001	0.78 (0.76, 0.80)	< 0.001	0.83 (0.82, 0.84)	< 0.001	0.80 (0.77, 0.82)	< 0.001
**Place of residence**												
Urban	1.00		1.00		1.00		1.00		1.00		1.00	
Rural	1.39 (1.33, 1.44)	< 0.001	1.04 (0.82, 1.32)	0.768	1.37 (1.30, 1.45)	< 0.001	1.08 (0.79, 1.49)	0.623	1.40 (1.32, 1.48)	< 0.001	1.00 (0.69, 1.43)	0.983
**Mother’s age**												
15–19	1.00		1.00		1.00		1.00		1.00		1.00	
20–24	0.75 (0.70, 0.81)	< 0.001	1.02 (0.71, 1.46)	0.914	0.72 (0.65, 0.79)	< 0.001	0.96 (0.60, 1.54)	0.868	0.80 (0.71, 0.89)	< 0.001	1.10 (0.63, 1.93)	0.729
25–29	0.73 (0.68, 0.79)	< 0.001	1.11 (0.78, 1.59)	0.556	0.72 (0.65, 0.79)	< 0.001	1.03 (0.65, 1.64)	0.899	0.75 (0.68, 0.84)	< 0.001	1.22 (0.71, 2.12)	0.475
30–34	0.71 (0.66, 0.76)	< 0.001	0.88 (0.60, 1.29)	0.502	0.69 (0.63, 0.76)	< 0.001	0.77 (0.46, 1.29)	0.329	0.73 (0.65, 0.82)	< 0.001	1.02 (0.57, 1.83)	0.956
35–39	0.74 (0.68, 0.80)	< 0.001	1.13 (0.76, 1.68)	0.540	0.73 (0.65, 0.81)	< 0.001	1.14 (0.68, 1.90)	0.612	0.76 (0.67, 0.85)	< 0.001	1.11 (0.60, 2.05)	0.751
40–44	0.78 (0.71, 0.85)	< 0.001	1.79 (1.17, 2.74)	0.007	0.71 (0.63, 0.80)	< 0.001	2.06 (1.18, 3.59)	0.010	0.86 (0.76, 0.98)	0.023	1.58 (0.81, 0.07)	0.178
45–49	0.97 (0.87, 1.09)	0.645	3.28 (1.96, 5.51)	< 0.001	0.94 (0.80, 1.10)	0.409	2.93 (1.50, 5.72)	0.002	1.02 (0.87, 1.21)	0.788	3.57 (1.57, 8.10)	0.002
**Mother’s educational level**												
None	1.00		1.00		1.00		1.00		1.00		1.00	
Primary	0.74 (0.71, 0.77)	< 0.001	1.83 (1.46, 2.29)	< 0.001	0.73 (0.69, 0.77)	< 0.001	1.88 (1.39, 2.54)	< 0.001	0.75 (0.71, 0.79)	< 0.001	1.79 (1.27, 2.50)	0.001
Secondary	0.57 (0.54, 0.59)	< 0.001	0.77 (0.57, 1.03)	0.079	0.56 (0.53, 0.60)	< 0.001	0.85 (0.58, 1.26)	0.421	0.57 (0.53, 0.61)	< 0.001	0.66 (0.42, 1.05)	0.077
Higher	0.27 (0.23, 0.32)	< 0.001	0.20 (0.07, 0.55)	0.002	0.27 (0.22, 0.35)	< 0.001	0.00	–	0.27 (0.21, 0.35)	< 0.001	0.39 (0.13, 1.15)	0.087
**Household wealth index**												
Poorest	1.00		1.00		1.00		1.00		1.00		1.00	
Poorer	0.98 (0.94, 1.03)	0.465	1.20 (0.94, 1.55)	0.149	0.96 (0.91, 1.02)	0.236	1.38 (0.98, 1.95)	0.064	1.01 (0.95, 1.07)	0.823	1.03 (0.71, 1.49)	0.886
Middle	0.86 (0.82, 0.91)	< 0.001	0.87 (0.65, 1.15)	0.331	0.84 (0.79, 0.90)	< 0.001	1.02 (0.70, 1.49)	0.925	0.89 (0.83, 0.95)	0.001	0.72 (0.47, 1.10)	0.131
Richer	0.77 (0.73, 0.81)	< 0.001	0.86 (0.63, 1.17)	0.335	0.76 (0.71, 0.81)	< 0.001	0.94 (0.62, 1.44)	0.785	0.78 (0.72, 0.84)	< 0.001	0.77 (0.49, 1.22)	0.269
Richest	0.56 (0.53, 0.60)	< 0.001	0.83 (0.58, 1.21)	0.336	0.58 (0.53, 0.62)	< 0.001	0.97 (0.59, 1.60)	0.903	0.55 (0.50, 0.60)	< 0.001	0.72 (0.41, 1.25)	0.238

*HR* Hazard ratio; *CI* Confidence Interval

## Discussion

In this study, the indicators used for measuring breastfeeding practices included time to initiation of breastfeeding after birth and duration of breastfeeding. We explored time to initiate breastfeeding and months of breastfeeding. Our findings showed disparities in the practices of breastfeeding across SSA countries. In addition, the differences in breastfeeding practices found in this study are consistent with some previous studies. In a meta-analysis of important breastfeeding variables using population data from 29 SSA nations, for example, beginning of breastfeeding within one hour after birth ranged from 37.8 to 69.3% [29]. Another study involving data from Burkina-Faso, DR Congo, Ethiopia, Kenya, Mali, Niger, Nigeria, Tanzania and Uganda reported pooled prevalence of initiation of breastfeeding within one hour after birth at 29.2 and 44.2% respectively. In studies conducted in Tanzania in 2010, the prevalence of breastfeeding within one hour after birth were 46.1 and 43.4% [30, 31]. These disparities among the countries could be due to differences in healthcare policies, maternal and child health care programmes, and healthcare seeking behaviour and practices respectively.

Other notable findings include female children having lower likelihood of consuming tinned, powdered/fresh milk, compared with male counterparts. This finding is consistent with a previous study in which female children had lower chance of receiving breast milk and fresh milk as a source of protein, compared with male counterparts [32]. On the other hand, female children were more likely to be put to breast within one hour after birth. Since breastfeeding within one hour after birth significantly reduces childhood mortality, this could also explain why male children have reportedly had higher risk of childhood mortality, compared with female counterparts [26]. The findings could also suggest that gender differences in feeding practices are likely to account for a significant proportion of male children disproportionately have worse survival outcomes relative to the female in SSA countries.

We found high infant mortality in Sierra Leone, Chad, Nigeria, Cote d’ Ivoire, Burkina Faso, and Mozambique respectively. According to the findings of our study, early breastfeeding initiation and increased breastfeeding duration were both associated with a lower risk of infant death. Maternal age was also linked to a higher risk of infant mortality. Higher educational attainment, on the other hand, was found to be a protective factor against infant mortality. These findings are in line with previous research findings [15, 26, 33]. Also, older women may have a higher level of parity, as childhood mortality has been commonly known to be higher among large households. Undoubtedly, higher educational level would help reduce childhood mortality, either by way of enhancing accessibility to health information which could positively influence healthcare seeking behaviour, or by the ability to pay for service delivery especially for individuals with no health insurance coverage, since higher education is known to enhance women’s participation in the labour force [34].

Breastfeeding patterns that included putting the child to breast within one hour of delivery and breastfeeding for a longer period of time reduced the risk of mortality in infants. The results are in line with earlier research reports [32, 35]. According to an epidemiological study, each additional month of breastfeeding resulted in a 6 % reduction in infant fatalities per 1000 live births [36]. Furthermore, our research found that breastfeeding for a longer period of time is associated with considerable survival benefits, with each month lowering the risk of mortality. Strong biological plausibility backs up the findings. Breastfeeding begins early, which lowers the necessity of prelacteal feeds, which have a significant risk of contamination, and breastfeeding appears to preserve and regulate the intestinal mucosa [37]. Colostrum, a component of breast milk, is high in immunological factors that protect against respiratory infections, enteric pathogens, and newborn sepsis [38, 39]. Intestinal maturation and epithelial recovery from infection are aided by breastfeeding. Early beginning has a protective impact due to some of the above direct effects as well as an effect on subsequent breastfeeding practices.

The findings of this study are useful in developing relevant indicators to measure the progress in SDGs especially those related to infant mortality, gender equality and childhood survival through early initiation and 24 months duration of breastfeeding. We found that female children are more likely to be put on breast within one hour than their male counterparts. In measuring SDG progress in gender equality, this can be a key indicator to determine whether male and female children are put to breast within one hour at the same rate. It is worrisome to note gender inequalities in early breastfeeding initiation among infants. In addition, the findings on infant mortality showed that many SSA countries are yet to achieve the less than 25 deaths per 1000 live births. The full coverage of early breastfeeding initiation and 24 months duration of breastfeeding are still practically lacking in SSA countries. The findings of this study have clearly contributed to further development of indicators to measures progress in SDGs.

### Strengths and limitations

The potential impact of selection bias is unlikely to affect the study findings due to the nationally representative big sample acquired using multi-stage probabilistic random sampling, which is one of the study’s key strengths. Furthermore, the study made use of DHS data, which were obtained using standardized questionnaires to assure consistency across geographies in SSA countries and to add to the evidence base. Data on the quantity of supplemental foods in other categories, however, was missing. As a result, it’s possible that males and females obtain food at the same time but in different amounts. Second, data on food intake was based on accounts from mothers. If mothers underreport discrepancies in feeding patterns, this could contribute to measurement inaccuracy. However, this would tend to conservatively bias the data, understating the degree of genuine nutritional disparities between males and females. Third, it’s possible that women are underreporting differences in breastfeeding and food access (a social desirability bias). Finally, because this is a cross-sectional study, we can’t prove causation, which raises issues when looking at the impacts of breastfeeding patterns on infant mortality risk, as it could lead to overestimation of the protective effects of early onset and length of breastfeeding.

## Conclusions

Our findings have brought to light, the need to give the promotion of early breastfeeding initiation and prolonged breastfeeding a priority with mothers. In spite of the fact that optimum breastfeeding is already part of WHO recommendations for newborn care, it is not a universal practice with about half of newborn babies in the world being breastfed in the first hour of life. Our findings have significant implications for programs and policies aimed at children’s health. This suggests that in resource-constrained situations, breastfeeding promotion programs should prioritize early beginning and breastfeeding duration. This is especially important in SSA countries where childhood mortality remains high. Based on our findings, we recommend future researchers to explore possible reasons while the female children are more likely put on the breast within one hour than their male counterparts.

## Data Availability

No additional data available.

## Abbreviations

BFHI: Baby friendly hospital initiative
DHS: Demographic and Health Survey
MDGs: The Millennium Development Goals
SDG-3: Third Sustainable Development Goal
SSA: Sub-Saharan Africa
WHO: World Health Organization
